# Complete Genome Sequence of Carotenoid-Producing Enterococcus gilvus CR1, Isolated from Raw Cow’s Milk

**DOI:** 10.1128/MRA.00988-18

**Published:** 2018-09-13

**Authors:** Shun Ohki, Tatsuro Hagi, Kazuma Nakano, Akino Shiroma, Hinako Tamotsu, Makiko Shimoji, Misuzu Shinzato, Noriko Ashimine, Maiko Minami, Tetsuhiro Nakanishi, Kuniko Teruya, Kazuhito Satou, Naoko Moriya, Miho Kobayashi, Masaru Nomura, Chise Suzuki, Takashi Hirano

**Affiliations:** a Okinawa Institute of Advanced Sciences, Uruma, Okinawa, Japan; b Institute of Livestock and Grassland Science, NARO, Tsukuba, Ibaraki, Japan; University of Maryland School of Medicine

## Abstract

Enterococcus gilvus CR1, isolated from raw cow’s milk, can produce carotenoids. The complete genome sequence of this strain was determined using the PacBio RS II platform. The assembly was found to contain a circular chromosome, including carotenoid biosynthesis genes, and comprises 2,863,043 bp, with a G+C content of 41.86% and three plasmids.

## Abstract (precis)

Enterococcus gilvus CR1, isolated from raw cow’s milk, can produce carotenoids. The complete genome sequence of this strain was determined using the PacBio RS II platform.

## ANNOUNCEMENT

The yellow-pigmented Enterococcus gilvus was first isolated from clinical specimens from humans in 2002 (1). In addition, E. gilvus strains were isolated from foods such as cheese and fermented sausages (2, 3). Therefore, these strains isolated from cheese (or milk) may aid cheese ripening by functioning as nonstarter lactic acid bacteria.

The yellow pigment produced by E. gilvus has been identified as diaponeurosporene, which is related to tolerance of hydrogen peroxide, low pH, bile acids, and lysozyme (4, 5). A study using E. gilvus CR1, isolated from raw cow's milk, showed that diaponeurosporene synthesis could be strongly induced under aerobic conditions, along with the upregulation of the gene expression level in the isoprenoid biosynthesis pathway and pyruvate dehydrogenase complex (6, 7).

The genome sequence is useful in clarifying the properties associated with fermentation and carotenoid biosynthesis regulation. To identify properties of CR1, the complete genome sequence was determined using single-molecule real-time (SMRT) technology (8). SMRT technology is a powerful tool for sequencing complete bacterial genomes with a highly repetitive sequence (9, 10).

CR1 was grown to early log phase in M17 medium (Difco Laboratories, Detroit, MI) supplemented with 0.5% glucose at 30°C under static condition. The genomic DNA was extracted as previously reported (11) and purified using a PowerClean DNA cleanup kit (Mo Bio Laboratories, Carlsbad, CA), which was followed by a 20-kb library construction for P6-C4 chemistry with shearing. Eight SMRT cells (240-min movie each) were used for sequencing on the RS II platform (Pacific Biosciences, Menlo Park, CA). *De novo* assembly was constructed using the hierarchical genome assembly process (HGAP) workflow (12) implemented in the SMRT analysis software v2.3.0 patch 5 (Pacific Biosciences) as an RS_HGAP_Assembly.2 protocol. In the protocol, we changed the following parameters from their defaults: compute minimum seed read length, unchecked; minimum seed read length, 10,000 bp; genome size, 4,000,000 bp; and target coverage, 15×. Resulting contigs were circularized using the Minimus2 pipeline from the AMOS v3.1.0 package (13) with its default parameters.

The genome sequence of CR1 contains one chromosome (2,863,043 bp, G+C content of 41.86%, and 1078× coverage) and three plasmids with sizes of 919,333 bp (G+C content of 42.94% and 935× coverage), 80,244 bp (G+C content of 35.03% and 1,434× coverage), and 82,704 bp (G+C content of 36.85% and 1,377× coverage). The PacBio RS II platform produced 1,665,885 reads with a mean length of 2,876 bp. The chromosome contains the isoprenoid biosynthesis pathway. The *spx* genes encoding transcriptional regulators involved in carotenoid biosynthesis (14) were found to be located on both the chromosome and the plasmids. Concerning fermentation properties, genes encoding peptidases and sugar metabolism, such as the phosphoenolpyruvate (PEP)-dependent phosphotransferase system, were also located on both the chromosome and the plasmids.

Further investigation into the CR1 genome will provide more insight into the regulation of carotenoid biosynthesis and milk fermentation.

### Data availability.

The complete genome sequence of Enterococcus gilvus CR1 has been deposited at DDBJ/ENA/GenBank under accession numbers CP030932 (chromosome), CP030933 (pCR1A), CP030934 (pCR1B), and CP030935 (pCR1C).
